# Dielectrophoresis of Amyloid-Beta Proteins as a Microfluidic Template for Alzheimer’s Research

**DOI:** 10.3390/ijms20143595

**Published:** 2019-07-23

**Authors:** Salman Ali Al-Ahdal, Aminuddin Bin Ahmad Kayani, Mohd Anuar Md Ali, Jun Yuan Chan, Talal Ali, Norah Adnan, Muhamad Ramdzan Buyong, Ervina Efzan Mhd Noor, Burhanuddin Yeop Majlis, Sharath Sriram

**Affiliations:** 1 Faculty of Engineering and Technology, Multimedia University, Melaka 75450, Malaysia; 2 Functional Materials and Microsystems Research Group and the Micro Nano Research Facility, RMIT University, Melbourne, Victoria 3001, Australia; 3 Institute of Microengineering and Nanoelectronics, Universiti Kebangsaan Malaysia, Bangi, Selangor 43600, Malaysia; 4 Faculty of Medicine, International University of Africa, Khartoum 12223, Sudan

**Keywords:** dielectrophoresis, microfabrication, bioelectric, microfluidics, microanalysis, lab-on-a-chip

## Abstract

We employed dielectrophoresis to a yeast cell suspension containing amyloid-beta proteins (Aβ) in a microfluidic environment. The Aβ was separated from the cells and characterized using the gradual dissolution of Aβ as a function of the applied dielectrophoretic parameters. We established the gradual dissolution of Aβ under specific dielectrophoretic parameters. Further, Aβ in the fibril form at the tip of the electrode dissolved at high frequency. This was perhaps due to the conductivity of the suspending medium changing according to the frequency, which resulted in a higher temperature at the tips of the electrodes, and consequently in the breakdown of the hydrogen bonds. However, those shaped as spheroidal monomers experienced a delay in the Aβ fibril transformation process. Yeast cells exposed to relatively low temperatures at the base of the electrode did not experience a positive or negative change in viability. The DEP microfluidic platform incorporating the integrated microtip electrode array was able to selectively manipulate the yeast cells and dissolve the Aβ to a controlled extent. We demonstrate suitable dielectrophoretic parameters to induce such manipulation, which is highly relevant for Aβ-related colloidal microfluidic research and could be applied to Alzheimer’s research in the future.

## 1. Introduction

**Figure 1:**
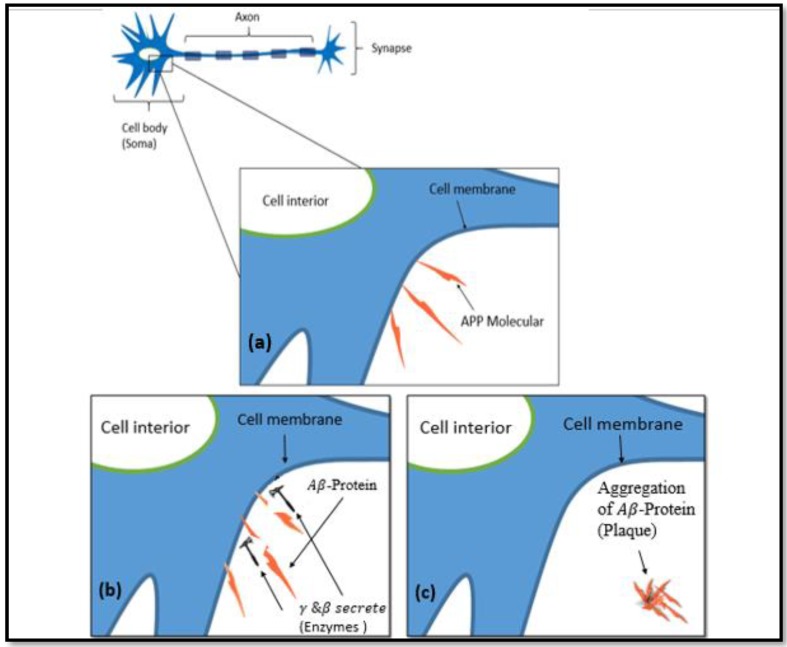
(**a**) Amyloid precursor protein (APP) is a primary membrane protein that is part of the cell membrane; (**b**) the cleaving of APP by incident enzymes; and (**c**) the aggregation of amyloid-beta (Aβ) to form a plaque.

The main clinical symptoms of Alzheimer’s disease (AD) include several cognitive disturbances, disorientation, inability to understand or recognize speech, and illusions [1]. The neurotic-anatomical examination of the patient shows a diffuse atrophy of the whole brain and distinctive variations of its internal structures, particularly on the cortical cell collections [2,3,4]. AD is measured by progressive disturbance in mental functions, with the main impact on memory efficiency [2,5,6]. Several dynamic processes, including synaptic damage [2,5,6,7], neuronal deterioration [8,9], disorder in neurotransmission [8,10], and disturbances in the activity of the neural network, have been identified as potential causes of the impairments. Morbid studies on AD have proven that these instabilities can occur within the brain regions [11]. AD is a deformation disease that targets the neurons of the brain [12,13] and is a common form of dementia that obliterates brain cells; hence, it affects the ability to think and damages the memory [11]. Accompanying AD is the loss of neurons and white matter, congophilic (amyloid) angiopathy, [14] inflammation, and oxidative damage [15]. Age is the main contributing factor underlying AD [9,13]. AD commonly targets populations that are about 60 years and older [9]. Aggressive propagation is the main characteristic of AD and affects the regions of memory inside the brain [13]. The common mechanism of disease propagation is amyloid-beta (Aβ) protein. Aβ is present in the interaction between the neurons in the brain. The protein is between 5 and 15 nm to a few micrometers in width and length, respectively [7]. Amyloid precursor protein (APP) is the primary membrane protein, as illustrated in Figure 1a, and is in charge of synapse construction [8], neural plasticity, and iron distribution [11]. The plaques are small elements of a special toxic protein that is generated from the surface of the cell membrane called Aβ. Moreover, a protein peptide occurs when β-secretase and γ-secretase cleave the APP, as depicted in Figure 1b. At this stage, the protein fragment is termed amyloid-beta (Aβ). Finally, a large number of protein fragments emerge to form a beta-amyloid plague (Figure 1c). Moreover, there is an amplification in Aβ assembly with a transformation in the APPs or the components involved in the proteolysis. There are hydrogen bonds in the Aβ assembly. These bonds form between the backbone oxygens and the amide hydrogens, which contribute to the well-established pattern of nucleation-dependent growth of amyloid fibrils [16]. The bonds can be broken as a result of excessive thermal heating [17].

Moreover, AD results in more plaques in particular brain regions, for example, the fornix and hippocampus. This project investigates new methods for researchers and sheds light on the destabilization of Aβ through the application of a microfluidic platform with the integration of dielectrophoresis (DEP).

### Basic Principle of Dielectrophoresis

DEP is a process of manipulating particle movement using non-uniform electric fields and it has wide bio-particle applications [18,19,20,21,22]. The force, magnitude, and direction of DEP are regulated by the relative particle polarization; therefore, the particles experience a force that either attracts the particles in the direction of the high electric field gradient region or a force that causes the particles to be repelled from those regions [23]. Conversely, DEP has been used for cell stimulation in order to manipulate, separate, fabricate, and analyze cell particles [24,25]. The DEP force that acts on a spherical particle is given by [26,27,28,29]:

(1) FDEP→=2πr3εmεoRe[fCM]∇E

where $\varepsilon_{o}$ = 8.854 × 10−12 F/m is the permittivity of the vacuum, *ε_m_* is the permittivity of the medium, *r* is the radius of the particle, Re[*f*_CM_] is the real part of the Clausius–Mossotti factor, and E is the applied electric field. If the Re[*f*_CM_] > 0, particles exposed will be attracted to regions of high electric field gradients and are said to experience positive DEP (pDEP). If Re[*f*_CM_] < 0, particles exposed will be repelled from regions of high electric field gradients and experience negative DEP (nDEP)

The *f*_CM_ depends on the dielectric and polarizability properties of the particles and the suspending medium and is expressed as [25,28]:

(2) $$f_{\mathrm{CM}}=\frac{\varepsilon_{p}^{*}-\varepsilon_{m}^{*}}{\varepsilon_{p}^{*}+2\varepsilon_{m}^{*}}$$

where $\varepsilon_{p}^{*}$ is the complex permittivity of the particle and $\varepsilon_{m}^{*}$ is the complex permittivity of the suspending medium and each is define as:

(3) $$\varepsilon^{*}=\varepsilon -j\frac{\sigma }{\omega }$$

where σ represents the electrical conductivity and ω represents the applied angular frequency.

The DEP process might be used to separate amyloid protein from nerve cells. Hypothetically, exposure of cells to electric fields and frequencies at definite parameters may eliminate AD, potentially without having an effect on nerve function. This elimination is a way of mitigating the resistance of Aβ so that the electric signal can flow through the neuron or it may be diluted.

A high electric field at the microelectrode generates electrothermal heating energy *W*, which affects the temperature of the surrounding suspending medium. The energy dissipated is governed by [30]:

*W* = *σE*^2^ (4)

in which *σ* is the suspending medium conductivity and *E* is the electrical field, as defined above.

The emitted electrothermal energy increases the surrounding medium temperature in the vicinity with the temperature increase Δθ given by:

(5) $$\Delta \theta =\frac{W}{m.C}$$

where *m* is the mass, and *C* is the specific heat capacity.

## 2. Results and Discussion

**Figure 2:**
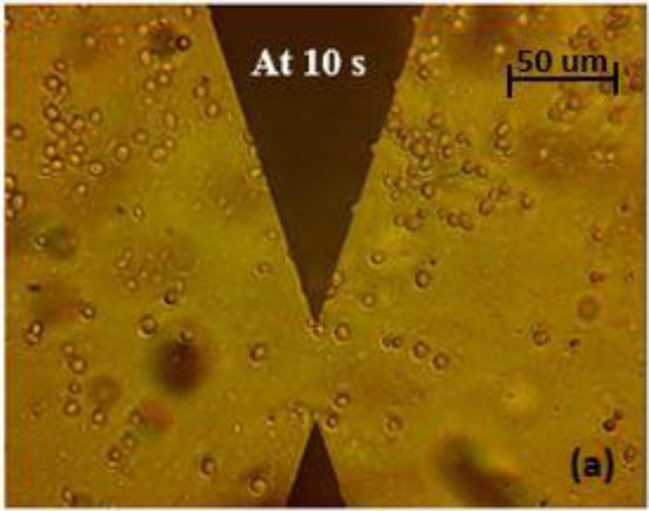
The uniform distribution of particles in a mixture of yeast cells and Aβ before applying dielectrophoresis (DEP) force.

Before DEP was applied, the mixture was injected into the platform. Since the parameters were not applied, the particles were uniformly distributed as shown in Figure 2.

A constant DEP voltage amplitude with selected variations of frequency from 10 MHz to 100 MHz at 5 V_pp_ was chosen. At higher electric fields, the DEP electrodes tended to experience excessive heating, which may have caused the Au and Cr to detach from the glass substrate [31]. Also, it has been observed that the smooth surface edges of the electrode could become jagged due to oxidation even though they were attached to glass. 

To avoid this phenomenon from occurring, we retained the amplitude at a relatively low level of 5 V_pp_ but managed to sweep the frequency from 10 MHz to 100 MHz without damaging the electrodes. In future works, it may be possible to incorporate better adhesion of the electrodes using alternative adhesion layers other than Cr or a more heat-resilient electrode such as titanium [31] and a substrate material such as PDMS [21]. However, when the frequency was increased from 10 MHz to 30 MHz, the particle suspension became exposed to the electric field gradient and the movement of the particles was attracted by other pairs of electrodes. 

**Figure 3:**
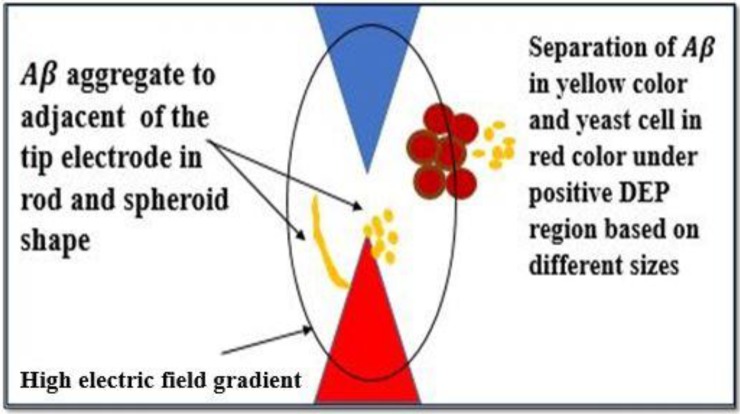
At 5 V_pp_ 60 MHz the particles gradually start to accumulate and form a package of cells at the electrode, indicating positive DEP (pDEP) for Aβ and negative DEP (nDEP) for yeast cells.

**Figure 4:**
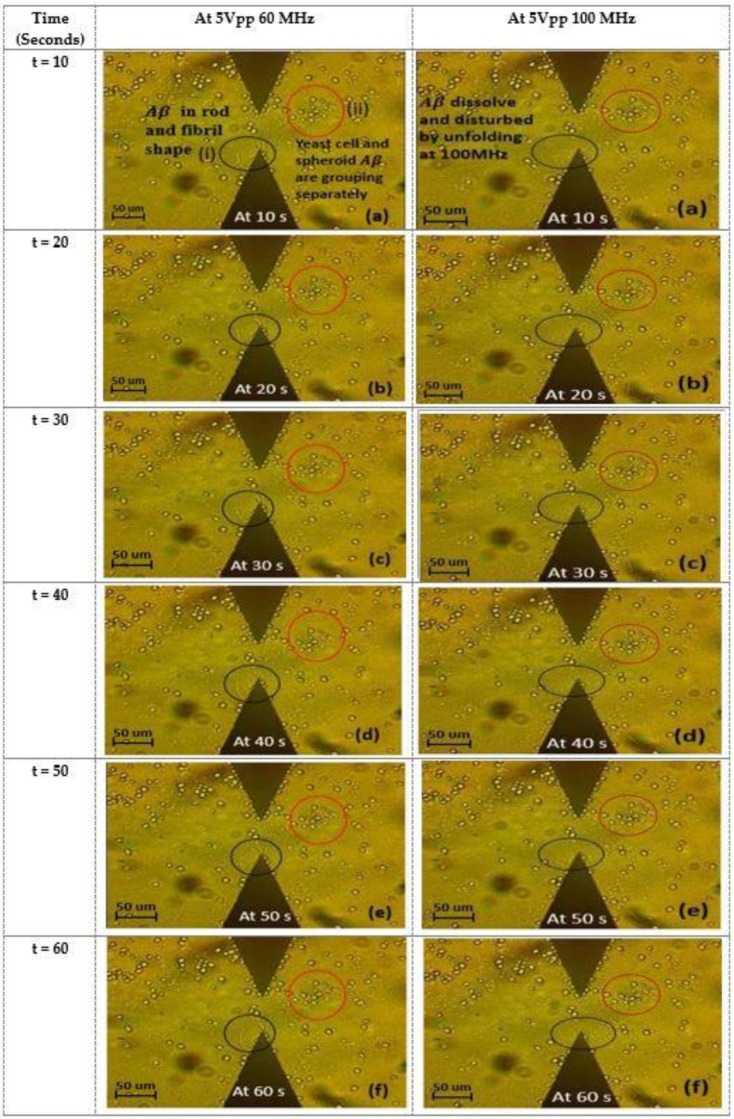
At 5 V_pp_ 60 MHz: (**a**) (i) t = 10 seconds the aggregation of Aβ to adjacent the electrode next to the tip, (**a**) (ii) t = 10 s showing initial separation of the mixture of yeast cells and Aβ (**b**–**f**) when t = 20–60 s there are the same results as in (**a**). At 5 V_pp_ 100 MHz (**a**–**f**) when t = 10–60 s the Aβ gradually dissolves by unfolding the Aβ fibril compared to at 5 V_pp_ 60 MHz.

When the frequency was increased from 30 MHz to 60 MHz, the particles started to accumulate and gradually formed a package of cells at the electrode, indicating pDEP for Aβ and nDEP for yeast cells. A positive DEP is a force that pulls the cells or particles toward the area surrounding the electrode, which contains a high electric field gradient. On the other hand, nDEP is the force that pulls the cells or particles away from the electrode, which contains a low electric field gradient as the frequency is increased to 60 MHz. The positive DEP force caused the Aβ to concentrate more in the high electric field gradient region adjacent to the electrode and next to the tip as shown in Figure 3 and Figure 4. Figure 3 and Figure 4 show the yeast cells and Aβ in the process of separating. The separation was due to the different DEP forces on the mixture and different sizes of the mixture. In addition, Aβ begins with monomers, dimers, and small oligomers joining to produce a pre-fibrillary oligomer nucleus.

**Figure 5:**
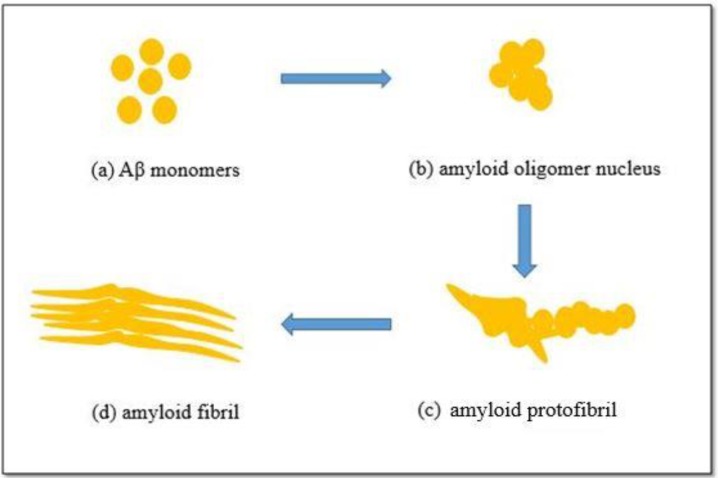
Stages in the formation of Aβ: (**a**) monomers, (**b**) the accumulation of small oligomers, (**c**) forming the amyloid protofibril, (**d**) Aβ in rod-shape, which is the final stage.

Monomers attach longitudinally to the core to generate elongated protofibril groups of strands. In this experiment, the Aβ used was synthesized in a powdered lyophilized form so, during dissolution of Aβ, it spent more time accumulating and formed fibrils that were rod-shaped agglomerations of monomers. Therefore, the time taken to transform the Aβ from the monomer stage (first stage) to the fibril rod stage (last stage) can vary. In other works, the time taken for Aβ to form fibril rods ranged from a few hours to a few days [32]. This time varied depending on the Aβ concentration and the pH of the suspension [32,33]. However, our platform had a fixed pH of approximately 7.5 [33,34]. Therefore, we can safely assume the period for transformation as mentioned above is accurate. At the time when the experimental results were captured, some monomers had formed fibril rods while others remained in the monomer or transitionary form. This is why some Aβ appeared spheroid while others were rod-shaped. The process of forming Aβ fibrils is shown in Figure 5.

**Figure 6:**
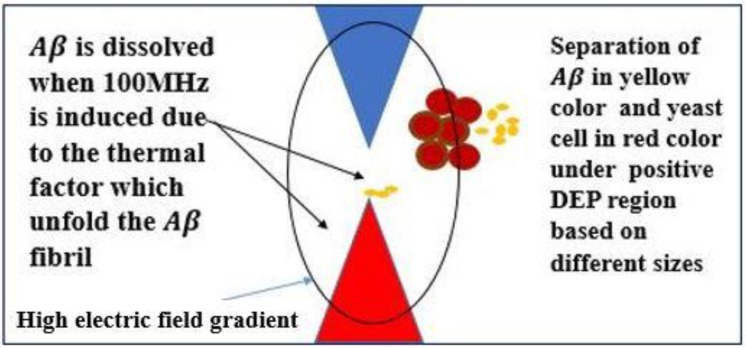
Schematic at 5 V_pp_ 100 MHz, at the tip of the electrode the temperature is increased at high frequency at high electric field 50 kV/m. Its effects on the structure of Aβ is explained above.

When the frequency was increased from 60 MHz to 100 MHz, at 60 s, the Aβ gradually dissolved (refer to Figure 4 and Figure 6). The Aβ at a high electric field gradient dissolved when it was in the fibril form; spheroidal-shaped monomers were delayed in the transformation process of forming visible Aβ fibrils.

This indicates that at high frequencies, the high temperature has the ability to break down the Aβ. Hydrogen bonds are present in the structure of proteins. The purpose of hydrogen bonds is to stabilize the structure of proteins. It was found that the DEP force with high frequency exposure generated thermal heating, which significantly destabilized the Aβ hydrogen bonds existing in the native structure of the protein. The stabilization of hydrogen bonds is critical as the DEP fields are known to produce thermal heating. In our experiments, the thermal heating was simulated as shown in Figure 4. Due to the unavailability of a thermal imaging camera, we simulated the thermal heating dissipation caused according to previously reported assumptions.

At the tip of the electrode where the electric field was around 50 kV/m, the temperature increased around 5.952 °C, as shown in Table 1. However, by tuning the frequency, the temperature increase can vary, perhaps due to changes in the properties of the suspending medium, particularly the conductivity [35] and elapsed time [36]. The conductivity may increase at a higher frequency, and subsequently increasing the temperature further may result in the breakdown of the Aβ. While at the electrode base with a low electric field region of 680 V/m (as shown in Table 1), the yeast cells were not affected by temperature. In yeast at a temperature of 37 °C, low magnitude electric fields with a high frequency have the ability to dissolve the Aβ protein by reversing the agglomeration process.

When the cells are re-suspended, the amyloid-beta proteins are, in fact, still in suspension. In our experiments, we found that the amyloid-beta produced fibril rods that were visible using microscope equipment. This represents an agglomeration of amyloid-beta as opposed to individual monomers that are indiscernible using the existing equipment. We proved that the fibril rods experience a separation from an agglomerated state and eventually disintegrate into smaller fibrils that are indiscernible under the microscope lens. This shows that from 60 MHz to 100 MHz, the DEP platform is able to separate and target amyloid-beta in the region of high electric field gradients.

## 3. Methodology

**Figure 7:**
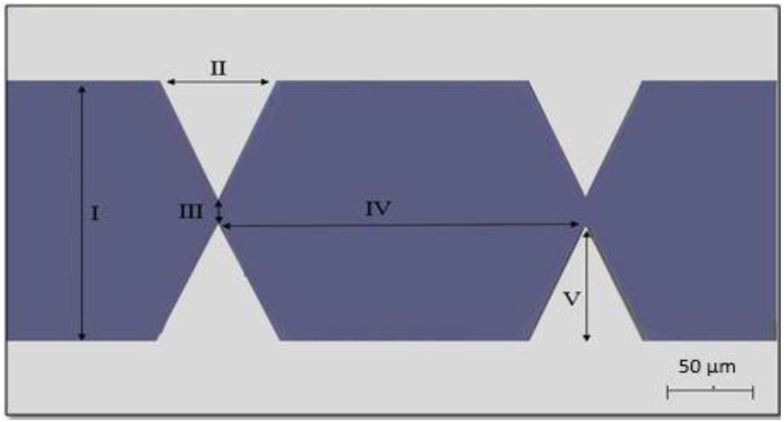
Dimensions of castellated microtip microelectrodes: (I) base to base = 300 µm, (II) base of the electrode = 100 µm, (III) spacing gap = 40 µm, (IV) tip to tip = 300 µm, and (V) the height of the electrode = 130 µm.

In our work, a microfluidic platform integrated with DEP microelectrodes was designed. The castellated microelectrode microtip pattern was designed using Autodesk Inventor (San Rafael, CA, USA) with dimensions shown in Figure 7.

### 3.1. Simulation

**Figure 8:**
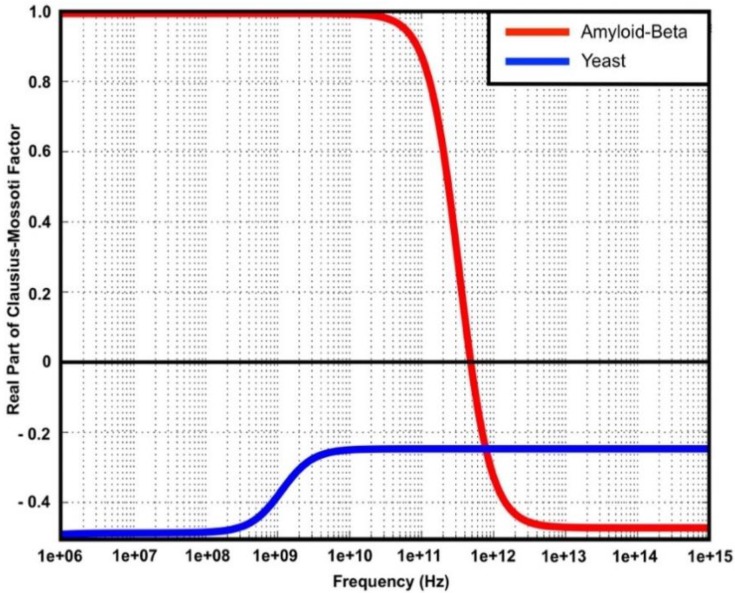
Re[*f*_CM_] of amyloid-beta protein and yeast cells in phosphate buffered saline (PBS) (50%).

**Figure 9:**
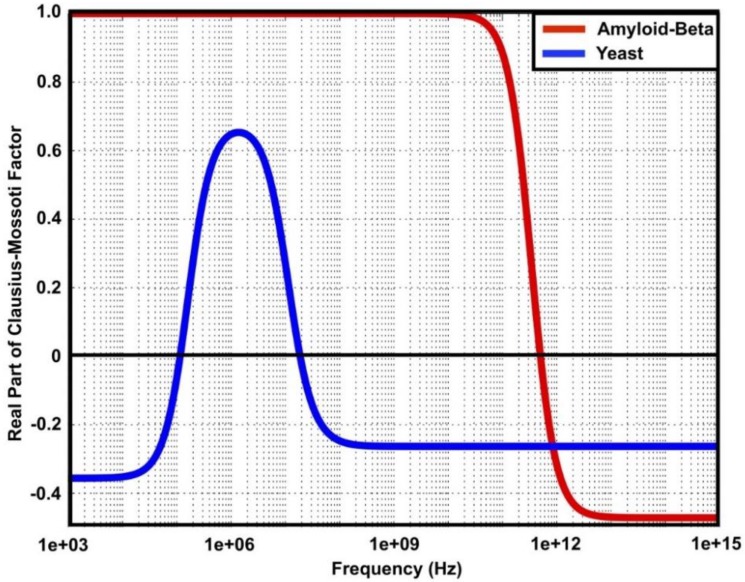
Re[*f*_CM_] of amyloid-beta protein and yeast cells in deionized water (DIW)**.**

Simulation was performed to predict particle behavior in two different media—phosphate buffered saline (PBS) 50% and deionized water (DIW)—under DEP force. The Re[*f*_CM_] was simulated using Matlab and the results for PBS 50% medium are shown in Figure 8 for frequencies from 1 MHz to 1 PHz. The parameters used for the simulation are shown in Table 2. It was found that the cross-over frequency for Aβ is located at *f_xo_* = 500 GHz, with a positive DEP experienced at lower frequencies. Yeast cells experienced negative DEP across the frequency range. In contrast, the results for DIW medium are shown in Figure 9. For frequencies from 1 KHz to 1 PHz, Aβ was located at *f_xo_* = 500 GHz with a positive DEP experienced at lower frequencies. The cross-over frequency existed around 100 kHz and 20 MHz for yeast cells with a positive DEP while a negative DEP was experienced at frequencies higher than 20 MHz.

**Figure 10:**
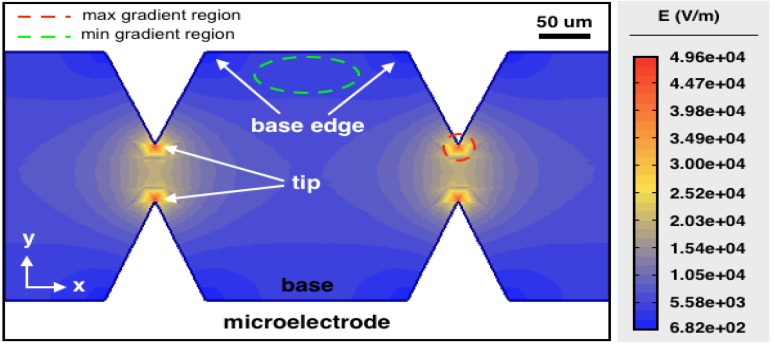
Simulation of electric field distribution where the dimensions used for the microelectrode design were as described in Figure 7.

Simulation was conducted to ascertain the fields generated by the DEP microelectrodes. The electric field distributions E were simulated using Agros2D 3.2, Czech Republic. The highest electric field of almost 50 kV/m was formed in the vicinity of the tips of the microelectrode, while the lowest of around 0.7 kV/m was formed at the edge of the base. The maximum gradient of the electric field was generated within a 10-µm radial distance from the microtip, shown as a red circle region in Figure 10 where the electric field dropped from 50 to 25 kV/m, i.e., the electric field gradient was around 2.5 × 10^9^ V/m. The minimum gradient was located within the base between the microtips, shown as a green line oval region in the figure where the electric field changes were almost negligible.

### 3.2. Fabrication Microelectrode Procedure

In this work, DEP was employed using yeast cells coated with amyloid beta protein. The substrate material was a glass slide as it is non-conductive and does not react with biological materials [28]. The glass slide (25 mm × 18 mm × 1 mm (L × W × H)) was cleansed using standard procedures, i.e., acetone-isopropanol and DIW, subsequently an ultrasonic bath prior to drying using a nitrogen air gun and exposure to an evaporative bake with a 120 °C hotplate for 5 min.

The microelectrode was fabricated using DC magnetron sputter where a 100-nm-thin layer of chromium (*Cr*) was deposited on a glass substrate to serve as an adhesion layer when a gold (*Au*) layer of thickness 150 nm was deposited onto it [41]. A positive photoresist AZ4620 (Microchemicals) was spun on the metal-deposited glass slides to coat them with 7 µm thickness photoresist. 

The glass slide was carefully coordinated with photomask inside a mask aligner for a 350 mJ/cm^2^ exposure energy of UV light. Following this, baking in a convection oven for 4 min at 90 °C was conducted [42]. Next, the remaining area of *Au*/*Cr* that was uncoated with photoresist was etched and removed by the application of a gold engraving solution and subsequent *Cr* etchant, up to a point where the glass slide clearly emerged [23]. To craft a translucent gold electrode, the seed layer was processed by boiling the slide in 18% HCl until the bubble of the seed layer disappeared. The slide was then rinsed with DIW and blow-dried using a nitrogen air gun. 

The microfluidic channel was fabricated from well-mixed polydimethylsiloxane (PDMS) elastomer and a curing agent (10:1), which was degassed in a vacuum chamber [43]. The mixture was then poured onto a microchannel and baked in a convective oven for 35 min at 100 °C. After the PDMS microchannel was peeled off from the mold and cooled to room temperature, holes for an inlet and an outlet were made using a biopsy puncher. The PDMS bonding surface was treated using a corona treater for 1 minute and then attached to an *Au/Cr* microelectrode substrate. 

### 3.3. Preparing the Yeast Cells and Aβ Protein Sample 

As yeast cells demonstrate a resemblance in various cell signaling and metabolic ways to nerve cells in the brain, they can be used to study Aβ aggregation, which is an important hallmark of AD [44]. Yeast was chosen as a model organism cell to characterize the DEP microfluidic device and electrode configuration, which is the initial step in further refining the platform. Yeast cells compared to mammalian cells do not have synapses, which is important for current transfer [44] and impedance measurements. However, since our experiments at this stage did not require such measurements, yeast cells could serve as a good model cell and this has been established in other research as well [44]. There were two stages to preparing the sample, which is a mixture of yeast cells and the Aβ protein. Initially, the yeast cell suspension was dissolved in DIW in a ratio of 1:3 (cell: DIW) instead of phosphate buffer saline (PBS) to provide a low conductivity suspending medium in order to observe the response of the mixture of yeast cells to the DEP force. Using DIW, electrically charged materials were removed, hence providing sole response of the cells to the DEP field with minimal influence from charged particles in the suspending medium. Even though PBS provides a more compatible environment for cells due to similar osmolality and ion concentrations as the human body, the dissolved salt ions do change the DEP field effects, which should be avoided. Secondly, the Aβ protein (BACHEM) came as a lyophilized white powder at room temperature. It was stored in the freezer at temperatures below −20 °C. Ammonium hydroxide (1.0%) was the solvent used to dissolve the lyophilized Aβ powder, trailed by a special kind of buffer known as phosphate buffered saline (1 × PBS) [45]. Ammonium hydroxide (1.0%) was added to the lyophilized Aβ powder. However, the peptide was not left for long in 1.0% ammonium hydroxide (NH_4_OH). It was, therefore, crucial to dilute the solution with phosphate buffered saline (1 × PBS) to a concentration of roughly 1 mg/mL [45,46,47]. The average size of Aβ was 5–15 nm in length and the Aβ used for the experiments came in that consistent dimension but in lyophilized form.

### 3.4. Experimental Procedure

During the experiments, the platform was placed on an inverted microscope (Amscope, Czech Republic) stage and a focusing lens was used to magnify the area of interest of the microelectrode. The small funnel was inserted into the inlet of the microfluidic in order to inject the specimen into the microchannel while the small tube and syringe was inserted in series into the outlet of the microfluidic. The power supply was connected to the platform with the parameters of 5 V_pp_ and range of frequency from 10 MHz to 100 MHz. The mixture of yeast and Aβ yielded different results at different operating DEP parameters. Ten microliters of the suspended sample of yeast cells and 10 µL of Aβ were mixed in a ratio of 1:1 and then injected into the microchannel through the inlet, which later was infused from the outlet manually via a syringe. Then, the voltages and frequencies were applied from a function generator (Regol, Beijing, China) to the microelectrodes.

## 4. Conclusions

In this experiment, we have differentiated particles with different sizes. When the size of the yeast cell was large in its range, the conductivity was reduced so the polarization of the particles was weak. With the low speeds involved to avoid leakage in the concertation of the sample, the flow of the medium was constant; the flow affects the output result of the DEP fields applied [21]. Additionally, it is a fast, low-voltage magnitude, and potentially low-cost method to extract measurements and to avoid hydrodynamic effects [19,48,49].

There was higher conductivity in the particle suspension with higher concentrations of yeast cells and Aβ relative to the suspending media. Hence, the DEP force required a higher voltage magnitude and suitable frequency for the effective separation and dissolution of Aβ [50]. Particles that undergo positive dielectrophoresis are less permeable than the surrounding medium [51].

In conclusion, the DEP platform involving the integrated microtip electrode array and microfluidic channel is able to selectively manipulate yeast and dissolve Aβ to a specific extent. The mixture of yeast cells and Aβ was characterized and it was found that at low frequencies ranging from 10 to 30 MHz and amplitude voltage of 5 V_pp_, the particles lacked a response due to the high conductivity of the sample. Higher electrical energy was required to penetrate the sample and induce a polarization in the suspension. When the frequency was increased, Aβ experienced positive DEP and subsequently yeast cells experienced nDEP. When the frequency was 60 MHz, the separation of yeast cells and Aβ occurred. Subsequently, the dissolution of Aβ was observed at 100 MHz.

We successfully demonstrated a viable template for microfluidic-based in vitro analyses and separation of Aβ from a model organism. The use of DEP microelectrodes for stimulating and manipulating AD-affected brain cells can be used using this same template. More importantly, this platform can be enhanced as a potential microfluidic template for biomedical research to further investigate Aβ or other types of proteins. 

## Figures and Tables

**Table 1:** Comparison between high and low electric field regions and their produced temperature.

Area	Temperature Increase, Δθ	Final Temperature (Initial = 27 °C)
At microtip (highest electric field region with 50 kV/m)	5.952 °C	32.925 °C
At electrode base (lowest electric field region with 680 V/m)	0.001 °C	27.001 °C

**Table 2:** Parameters for the Clausius–Mossoti Factor Calculation.

Particle	Parameter	Value	Reference
**Aβ Protein**	protein size, *r_Aβ_*	5 to 15 (nm)	[7,37]
	protein conductivity, *σ_Aβ_*	3 × 10^3^ (S/m)	
	core permittivity, *ε_Aβ_*	(2 to 4) *ε*_o_ (F/m)	
	interface permittivity, *ε_int_*	(10 to 20) *ε*_o_ (F/m)	
**Yeast**	membrane thickness, *d_mem_*	8 (nm)	[38]
	cell wall thickness, *d_wall_*	220 (nm)	
	cell radius, *r_yeast_*	3 (μm)	
	cytoplasm conductivity, *σ_cyt_*	0.2 (S/m)	
	membrane conductivity, *σ_mem_*	2.5 × 10^−8^ (S/m)	
	cell wall conductivity, *σ_wall_*	1.4 × 10^−3^ (S/m)	
	cytoplasm permittivity, *ε_cyt_*	50 *ε*_o_ (F/m)	
	membrane permittivity, *ε_mem_*	6 *ε*_o_ (F/m)	
	cell wall permittivity, *ε_wall_*	60 *ε*_o_ (F/m)	
**PBS (50%)**	medium conductivity, *σ_m_*	6 (S/m)	[39,40]
	medium permittivity, *ε_m_*	80 *ε*_o_ (F/m)	
**DIW**	Medium conductivity, *σ_m_*	0.01(S/m)	[41]
	Medium permittivity, *ε_m_*	78 *ε*_o_ (F/m)	

## References

[1] Möller H.J., Graeber M.B. (1998). The Case Described by Alois Alzheimer In 1911. Historical and Conceptual Perspectives Based on The Clinical Record And Neurohistological Sections. Eur. Arch. Psychiatry Clin. Neurosci..

[2] Aldavert-Vera L., Huguet G., Costa-Miserachs D., Ortiz S.P., Kádár E., Morgado-Bernal I., Segura-Torres P. (2013). Intracranial Self-Stimulation Facilitates Active-Avoidance Retention and Induces Expression of C-Fos and Nurrl in Rat Brain Memory Systems. Behav. Brain Res..

[3] Huguet G., Aldavert-Vera L., Kádár E., Peña de Ortiz S., Morgado-Bernal I., Segura-Torres P. (2009). Intracranial Self-Stimulation to The Lateral Hypothalamus, a Memory Improving Treatment, Results in Hippocampal Changes in Gene Expression. Neuroscience.

[4] Klooster D.C.W., de Louw A.J.A., Aldenkamp A.P., Besseling R.M.H. (2016). Neuroscience and Biobehavioral Reviews Technical Aspects of Neurostimulation: Focus on Equipment, Electric Field Modeling, and Stimulation Protocols. Neurosci. Biobehav. Rev..

[5] Wimo A., Prince M. (2010). World Alzheimer Report 2010 The Global Economic Impact of Dementia. https://www.alz.co.uk/research/files/WorldAlzheimerReport2010ExecutiveSummary.pdf.

[6] Prince M., Knapp M., Guerchet M., McCrone P., Prina M., Comas-Herrera A., Wittenberg R., Adelaja B., Hu B., King D. (2014). Dementia UK: Second Edition—Overview. http://eprints.lse.ac.uk/59437/1/Dementia_UK_Second_edition_-_Overview.pdf.

[7] Leinenga G., Götz J. (2015). Scanning Ultrasound Removes Amyloid-β and Restores Memory in an Alzheimer’ s Disease Mouse Model. Sci. Transl. Med..

[8] Priller C., Bauer T., Mitteregger G., Krebs B., Kretzschmar H.A., Herms J. (2006). Synapse Formation and Function Is Modulated by the Amyloid Precursor Protein. J. Neurosci..

[9] Querfurth H.W., Laferla F.M. (2010). Alzheimer’s Disease. N. Engl. J. Med..

[10] Gendron T.F., Petrucelli L. (2009). The Role of Tau in Neurodegeneration. Mol. Neurodegener..

[11] Laxton A.W., Tang-Wai D.F., McAndrews M.P., Zumsteg D., Wennberg R., Keren R., Wherrett J., Naglie G., Hamani C., Smith G.S. (2010). A Phase I Trial of Deep Brain Stimulation of Memory Circuits in Alzheimer’ s Disease. Ann. Neurol..

[12] Ren Y. (2015). Towards Brain-on-a-Chip: Microfluidic and Microelectrode Array Platforms for Morphological and Electrophysiological Observations on the Propagation of Alzheimer’s Disease. Ph.D. Thesis.

[13] Sabbagh J.J., Kinney J.W., Cummings J.L. (2013). Animal systems in The Development of Treatments for Alzheimer’s Disease: Challenges, Methods, and Implications. Neurobiol. Aging.

[14] Exley C., Esiri M.M. (2006). Severe Cerebral Congophilic Angiopathy Coincident with Increased Brain Aluminium in a Resident of Camelford, Cornwall, UK. J. Neurol. Neurosurg. Psychiatry.

[15] Patel V.P., Chu C.T. (2011). Nuclear Transport, Oxidative Stress, and Neurodegeneration. Int. J. Clin. Exp. Pathol..

[16] Hubbard R.E., Haider M.K. (2010). Hydrogen Bonds in Proteins: Role and Strength. Encycl. Life Sci..

[17] Cooper A. (2000). Heat Capacity of Hydrogen-Bonded Networks: An Alternative View of Protein Folding Thermodynamics. Biophys. Chem..

[18] Khoshmanesh K., Nahavandi S., Baratchi S., Mitchell A., Kalantar-zadeh K. (2011). Biosensors and Bioelectronics Dielectrophoretic Platforms for Bio-microfluidic Systems. Biosens. Bioelectron..

[19] Khoshmanesh K., Zhang C., Campbell J.L., Kayani A.A., Nahavandi S., Mitchell A., Kalantar-Zadeh K. (2010). Dielectrophoretically Assembled Particles: Feasibility for Optofluidic Systems. Microfluid. Nanofluidics.

[20] Ali M.A.M., Ostrikov K., Khalid F.A., Majlis B.Y., Kayani A.A. (2016). Active Bioparticle Manipulation in Microfluidic Systems. RSC Adv..

[21] Kung Y.C., Huang K.W., Chong W., Chiou P.Y. (2016). Tunnel Dielectrophoresis for Tunable, Single-Stream Cell Focusing in Physiological Buffers in High-Speed Microfluidic Flows. Small.

[22] Kung Y.C., Huang K.W., Fan Y.J., Chiou P.Y. (2015). Fabrication of 3D High Aspect Ratio PDMS Microfluidic Networks with a Hybrid stamp. Lab Chip.

[23] Waheed W., Alazzam A., Abu-Nada E., Khashan S., Abutayeh M. (2018). A Microfluidics Device for 3D Switching of Microparticles Using Dielectrophoresis. J. Electrostat..

[24] Mohamad A.S., Hamzah R., Hoettges K.F., Hughes M.P. (2017). A Dielectrophoresis-Impedance Method for Protein Detection and Analysis. AIP Adv..

[25] Ali M.A.M., Majlis B.Y., Azman Z.N., Kayani A.A. Cell-Cell Contact Configurations by Dielectrophoresis for Electrofusion: Study on Directions, Stability and Dielectric Heating Effect. Proceedings of the 2016 IEEE EMBS Conference on Biomedical Engineering and Sciences (IECBES).

[26] Kayani A.A., Khoshmanesh K., Ward S.A., Mitchell A., Kalantar-zadeh K. (2012). Optofluidics Incorporating Actively Controlled Micro- and Nano-Particles Optofluidics Incorporating Actively Controlled. Biomicrofluidics.

[27] Kayani A.A., Chrimes A.F., Khoshmanesh K., Sivan V., Zeller E., Kalantar-zadeh K., Mitchell A. (2011). Interaction of Guided Light in Rib Polymer Waveguides with Dielectrophoretically Controlled Nanoparticles. Microfluid. Nanofluidics.

[28] Chrimes A.F., Kayani A.A., Khoshmanesh K., Stoddart P.R., Mulvaney P., Mitchell A., Kalantar-Zadeh K. (2010). Dielectrophoresis-Raman Spectroscopy System for Analysing Suspended Nanoparticles. Lab Chip.

[29] Yi P., Kayani A.A., Chrimes A.F., Ghorbani K., Nahavandi S., Kalantar-zadeh K., Khoshmanesh K. (2012). Thermal Analysis of Nanofluids in Microfluidics Using an Infrared Camera. Lab Chip.

[30] Ramos A., Morgan H., Green N.G., Castellanos A. (1998). AC Electrokinetics: A Review of Forces in Microelectrode Structures. J. Phys. D Appl. Phys..

[31] Chan J.Y., Ahmad Kayani A.B., Md Ali M.A., Kok C.K., Yeop Majlis B., Hoe S.L.L., Marzuki M., Khoo A.S., Ostrikov K.K., Ataur Rahman M. (2018). Dielectrophoresis-Based Microfluidic Platforms for Cancer Diagnostics. Biomicrofluidics.

[32] Jeanmonod D.J., Suzuki R.K., Hrabovsky M. (2018). Supramolecular Organization of Amyloid Fibrils. Intech Open.

[33] Schleeger M., Deckert-Gaudig T., Deckert V., Velikov K.P., Koenderink G., Bonn M. (2013). Amyloids: From Molecular Structure to Mechanical Properties. Polymer.

[34] Domigan L., Andersen K.B., Sasso L., Dimaki M., Svendsen W.E., Gerrard J.A., Castillo-León J. (2013). Dielectrophoretic Manipulation and Solubility of Protein Nanofibrils Formed from Crude Crystallins. Electrophoresis.

[35] Fischer H.E. (1966). Scholars’ Mine an AC Method of Measuring the Conductivity of Dielectric Liquids. Master’s Thesis.

[36] Boulanger L. (1998). Observations on Variations in Electrical Conductivity of Pure Demineralized Water: Modification (‘Activation’) of Conductivity by Low-Frequency, Low-Level Alternativing Electric Fields. Int. J. Biometeorol..

[37] Gitlin I., Carbeck J.D., Whitesides G.M. (2006). Proteins Why Are Proteins Charged? Networks of Charge—Charge Interactions in Proteins Measured by Charge Ladders and Capillary Electrophoresis Angewandte. Angew. Chem. Int. Ed..

[38] Patel S., Showers D., Vedantam P., Tzeng T.R., Qian S., Xuan X. (2012). Microfluidic Separation of Live and Dead Yeast Cells Using Reservoir-Based Dielectrophoresis. Biomicrofluidics.

[39] Zheng Y., Nguyen J., Wang C., Sun Y. (2013). Electrical Measurement of Red Blood Cell Deformability on a Microfluidic Device. Lab Chip.

[40] Chaparro C.V., Herrera L.V., Meléndez A.M., Miranda D.A. (2016). Considerations on electrical impedance measurements of electrolyte solutions in a four-electrode cell. J. Phys. Conf. Ser..

[41] Ali M.A.M., Kayani A.B.A., Yeo L.Y., Chrimes A.F., Ahmad M.Z., Ostrikov K.K., Majlis B.Y. (2018). Microfluidic Dielectrophoretic Cell Manipulation Towards Stable Cell Contact Assemblies. Biomed. Microdevices.

[42] Yafouz B., Kadri N.A., Ibrahim F. (2013). Microarray Dot Electrodes Utilizing Dielectrophoresis for Cell Characterization. Sensors.

[43] Alhammadi F., Waheed W., El-Khasawneh B., Alazzam A. (2018). Continuous-Flow Cell Dipping and Medium Exchange in a Microdevice Using Dielectrophoresis. Micromachines.

[44] Moosavi B., Mousavi B., Macreadie I.G. (2015). Yeast Model of Amyloid-β and Tau Aggregation in Alzheimer’s Disease. J. Alzheimer’s Dis..

[45] Johnson R.D., Schauerte J.A., Wisser K.C., Gafni A., Steel D.G. (2011). Direct Observation of Single Amyloid-β(1-40) Oligomers on Live Cells: Binding and Growth at Physiological Concentrations. PLoS ONE.

[46] Ryan T.M., Caine J., Mertens H.D., Kirby N., Nigro J., Breheney K., Waddington L.J., Streltsov V.A., Curtain C., Masters C.L. (2013). Ammonium Hydroxide Treatment of Aβ Produces an Aggregate Free Solution Suitable for Biophysical and Cell Culture Characterization. PeerJ.

[47] Hellstrand E., Boland B., Walsh D.M., Linse S. (2010). Amyloid Protein Aggregation Produces Highly Reproducible Kinetic Data and Occurs by a Two-Phase Process. ACS Chem. Neurosci..

[48] Kayani A.A., Khoshmanesh K., Nguyen T.G., Kostovski G., Chrimes A.F., Nasabi M., Heller D.A., Mitchell A., Kalantar-zadeh K. (2012). Dynamic Manipulation of Modes in an Optical Waveguide Using Dielectrophoresis. Electrophoresis.

[49] Khoshmanesh K., Tovar-Lopez F.J., Baratchi S., Zhang C., Kayani A.A., Chrimes A.F., Nahavandi S., Wlodkowic D., Mitchell A., Kalantar-zadeh K. (2011). Dielectrophoresis of Micro/Nano Particles Using Curved Microelectrodes. Proc. SPIE Int. Soc. Opt. Eng..

[50] Wang P., Chang H. (2006). Bacteria Capture, Concentration and Detection by Alternating Current Dielectrophoresis and Self-Assembly of Dispersed Single-Wall Carbon Nanotubes. Electrophoresis.

[51] Staton S.J.R., Jones P.V., Ku G., Gilman S.D., Kheterpal I., Hayes M.A. (2012). Manipulation and Capture of Aβ Amyloid Fibrils and Monomers by DC Insulator Gradient Dielectrophoresis (DC-iGDEP). Analyst.

